# Real-World Evidence That As-Needed Dosing with Bimekizumab in Patients with Psoriasis Is Safe and Effective over Time

**DOI:** 10.3390/jpm16050234

**Published:** 2026-04-27

**Authors:** Carlota Abbad-Jaime De Aragón, María Davo-Mogica, Pablo de la Cruz-Anaya, Emilio Berna-Rico, Inés Díaz-Ruiz, Inés Perales-Sánchez, Nicholas D. Brownstone, Pedro Jaén, Lluis Puig, Andrew Blauvelt, Alvaro González-Cantero

**Affiliations:** 1 Department of Dermatology, Hospital Universitario Ramón y Cajal, Instituto Ramon Y Cajal de Investigación Sanitaria (IRYCIS), 28034 Madrid, Spain; carlotababbad@gmail.com (C.A.-J.D.A.);; 2 Inflammatory Disease and Psoriasis Unit, Grupo Pedro Jaén, 28006 Madrid, Spain; 3 Faculty of Medicine, Universidad Francisco de Vitoria, 28223 Madrid, Spain; 4 Department of Vascular Physiopathology, Hospital Nacional de Parapléjicos, Servicio de Salud de Castilla-La Mancha (SESCAM), 45071 Toledo, Spain; 5 Department of Vascular Physiopathology, Hospital Nacional de Parapléjicos, Instituto de Investigación Sanitaria de Castilla-La Mancha (IDISCAM), 45071 Toledo, Spain; 6 Department of Dermatology, Icahn School of Medicine at Mount Sinai, New York, NY 10029, USA; 7 Department of Dermatology, Hospital de la Santa Creu i Sant Pau, Institut d’Investigació Biomèdica Sant Pau (IIB SANT PAU), Universitat Autònoma de Barcelona, 08041 Barcelona, Spain; 8 Blauvelt Consulting, LLC, Annapolis, MD 21401, USA

**Keywords:** psoriasis, bimekizumab, as-needed, on-demand, off-label

## Abstract

**Background/Objectives**: Despite substantial progress in the management of psoriasis, evidence on as-needed dosing strategies for biologic therapies remains scarce. In this context, the present study aimed to assess the effectiveness of a previously defined as-needed bimekizumab (BKZ) dosing regimen in a larger cohort of patients with psoriasis, as well as to identify clinical factors associated with treatment response. **Methods**: In this retrospective study, medical records of 64 patients with moderate-to-severe psoriasis treated with BKZ between May 2023 and November 2025 at a specialized psoriasis unit in Madrid, Spain, were reviewed. Patients followed an off-label, as-needed dosing regimen, consisting of two initial 320 mg doses at Weeks 0 and 4, with subsequent administrations only upon loss of a PASI 90 response. The primary outcome was the proportion of patients achieving and maintaining optimal disease control (PASI 90) over time. The duration of treatment effect was defined as the interval between the second dose and loss of PASI90 in the absence of further treatment. Safety outcomes were also evaluated. **Results**: A total of 59 out of 64 patients achieved a PASI 90 response after the initial two BKZ doses, and all maintained disease control with the as-needed dosing strategy over time. On average, patients received approximately one-third of the doses expected under the standard dosing regimen. The mean duration of treatment effect following the second dose was approximately 24 weeks. Systemic and bio-naïve patients presented the longest treatment effect duration under the as-needed dosing regimen. Oral candidiasis was reported in two patients and resolved without complications. **Conclusions**: This study reinforces previous evidence supporting the effectiveness of an as-needed BKZ dosing strategy, particularly in patients naïve to systemic and biologic therapies for psoriasis. Nevertheless, larger prospective studies are required to confirm these findings.

## 1. Introduction

Psoriasis is a chronic immune-mediated inflammatory disease driven by IL-23/Th17 pathway dysregulation, affecting 2–3% of the world’s population [1,2]. The introduction of new-generation biologic agents targeting this pathway has revolutionized psoriasis management, achieving high and sustained efficacy with standard dosing regimens [3]. Current treatment strategies, however, are associated with substantial long-term healthcare costs [4]. To address this issue, recent studies have explored off-label dosing regimens, which may significantly reduce costs and enhance individual risk–benefit considerations [5]. Most available real-world evidence on dose reduction strategies has been derived from experience with anti-TNF and anti-IL-12/23 agents [5], and more recently, IL-17 and IL-23 inhibitors [6,7], although there is a relative lack of data for alternative dosing strategies for anti-IL-17 therapies. Despite being next-generation biologics, outcomes vary significantly across drug classes. IL-23 inhibitors demonstrate superior skin clearance and sustained therapeutic durability, likely due to their modulation of pathogenic T-cell memory. Furthermore, IL-17, particularly IL-17A inhibitors, yield more heterogeneous results, suggesting that clinical stability in this class appears highly sensitive to dose spacing. Notably, there is still a lack of robust information regarding dual anti-IL-17A/F inhibitors and their performance under extended-interval regimens. Despite these emerging data, implementation of alternative dosing strategies in routine practice remains hampered by a lack of standardized protocols and long-term longitudinal data. Bimekizumab (BKZ), a monoclonal antibody that selectively inhibits both IL-17A/F, represents the latest therapeutic advancement for moderate-to-severe plaque psoriasis. Its dual mechanism of action achieves a more profound suppression of inflammation than traditional IL-17A inhibitors, yet its potential for flexible dosing in clinical practice remains under-explored. To address this gap, we previously reported a retrospective case series of 28 patients treated with BKZ using an individualized, as-needed dosing strategy. All patients received the first two BKZ doses (at Weeks 0 and 4) and then were only dosed again if the response fell below PASI 90. All patients achieved and sustained PASI 90 responses with this as-needed dosing strategy [8]. Herein we expand upon our preliminary findings by increasing both the sample size and the follow-up duration in patients with psoriasis receiving as-needed BKZ dosing, aiming to assess the durability of this therapeutic approach and to identify clinical predictors of success. This dosing strategy is consistent with a personalized treatment approach with BZK by optimizing a regimen based on patient-specific needs.

## 2. Materials and Methods

### 2.1. Study Population

Adult patients with moderate-to-severe psoriasis treated with BKZ in routine clinical practice, who have received BKZ doses at weeks 0 and 4, and with at least one in-person follow-up assessment were included.

### 2.2. Treatment Strategy

This retrospective case series is an extension of a previously published cohort evaluating an off-label, as-needed dosing strategy of BKZ in patients with moderate-to-severe psoriasis [8]. Medical records of consecutive patients treated at the Psoriasis Unit of the Grupo Pedro Jaen (Madrid, Spain) were evaluated from May 2023 to November 2025. In the present analysis, a total of 64 patients were included. All patients received two 320 mg BKZ doses, one at Week 0 and one at Week 4. Subsequent 320 mg doses were administered only if a patient dropped below a PASI 90 response on a routine follow-up visit. Concomitant topical therapies were not permitted during the study period. No patients deviated from the as-needed dosing strategy, although minor variations inherent to routine clinical practice may have occurred. All patients provided written informed consent.

### 2.3. Follow-Up and Outcomes

The follow-up strategy was the same as the one described previously [8]. Patients underwent both in-person evaluations and online monitoring. Patients were assessed in person at weeks 0, 4 and every 4 months thereafter. Additionally, in-person evaluations were performed whenever a patient required a new dose of BKZ. During these visits, the PASI score was formally assessed by a dermatologist.

Concurrently, following the initiation of BKZ treatment at Weeks 0 and 4, all patients were subject to telephone or e-mail follow-up evaluations every 4 weeks to monitor therapeutic progress. During these remote assessments, the clinical team utilized standardized questions to verify the maintenance of response. Patients were instructed to contact the clinic if they perceived any loss of the optimal skin clearance achieved during the induction phase.

In the present study, the threshold for administering a new dose of BKZ was defined as the clinically confirmed loss of PASI 90 response. This endpoint was selected in accordance with previously established protocols [9] to reflect evolving therapeutic standards favoring high levels of skin clearance. Within this methodological framework, PASI 90 was utilized as the primary clinically meaningful target, representing a balance between the stringency of PASI 100 and the conventional PASI 75 benchmark in a real-world setting. A new dose was only administered if the formal assessment confirmed a drop below the PASI 90 threshold.

Treatment effect duration was defined as the interval (in weeks) from the second BKZ dose at Week 4 to the loss of PASI 90 in the absence of treatment. Safety outcomes were also assessed.

### 2.4. Statistical Analysis

Values are reported as mean (standard deviation [SD]) for parametric variables, median (interquartile range [IQR]) for nonparametric variables, and *n* (%) for categorical variables. Normality was evaluated using skewness, kurtosis, and histogram plots. The distribution of treatment effect duration in all patients was graphically represented by a dispersion plot. Correlation analyses were performed using Pearson or Spearman correlation to explore associations between treatment effect duration and clinical variables. Between-group comparisons according to the number of previous systemic treatments were performed using Student’s *t*-test or ANOVA for normally distributed variables, and the Mann–Whitney U or Kruskal–Wallis test for non-normally distributed variables. Categorical variables were compared using the chi-square test or Fisher’s exact test.

A multivariable linear regression model was used to identify independent predictors of treatment effect duration. Variables included in the model were selected based on clinical relevance and results from univariate analyses. A two-sided *p*-value < 0.05 was considered statistically significant. Statistical analyses were performed using R.

## 3. Results

This case series included 64 patients (42 males, 22 females) with psoriasis. At the start of BKZ treatment, mean age was 42.4 ± 16.2 years, mean body mass index (BMI) was 25.1 ± 3.9 kg/m^2^, and mean psoriasis duration 14.7 ± 11.8 years. The Mean baseline Psoriasis Area Severity Index (PASI) was 7.1 ± 4.8. Previous conventional systemic therapy had been received by 32.8% (N = 21) of patients, and 31.3% previously received biologic therapy (N = 20). Regarding cardiovascular risk factors, dyslipidemia was reported in 37.5% of patients, hypertension in 10.9%, and 14.1% were active smokers. Psoriatic arthritis was present in 10.9% of patients (Table 1).

**Figure 1:**
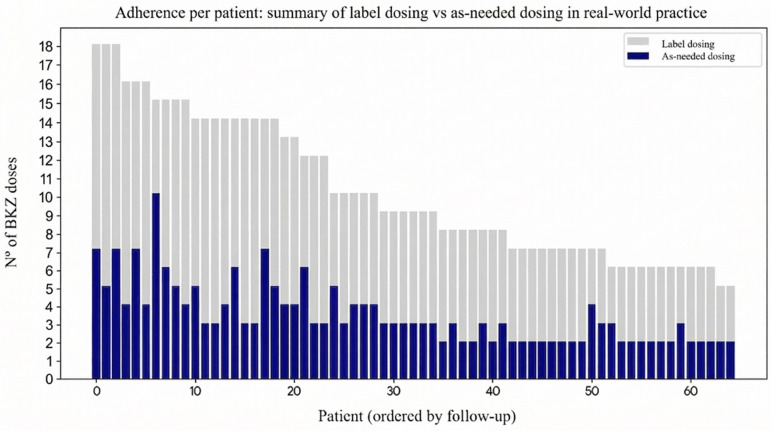
As-needed dosing of moderate-to-severe psoriasis patients with bimekizumab. **Legend**: BKZ: Bimekizumab. Blue bars: the number of BKZ doses each patient received. In grey: the number of BKZ doses they received if the standard label had been followed.

The primary study outcome was the maintenance of a PASI 90 response under an as-needed dosing strategy; the median (IQR) follow-up was 12 [7–19] months. PASI 90 response was achieved by 63.5% of patients at week 4, after one dose. At week 8, during the routine follow-up call—by which time patients had completed the two-dose induction regimen—59 of 64 patients (92.2%) had achieved PASI 90, and this response was sustained throughout their respective follow-up periods. Moreover (41 of 64 patients), 64% maintained a PASI ≤ 1 and 52 of 64 patients (81%) PASI ≤ 2 throughout the follow-up. Mean treatment effect duration after the two first BKZ doses was 25.6 ± 7.6 weeks (6.4 ± 1.9 months). Figure 1 shows the number of BKZ doses received (blue), versus the doses that each patient would have received if the on-label dosing of BKZ had been followed (grey). Over the study period, patients received on average approximately one-third of the doses that would have been expected if on-label dosing had been followed (3.46 ± 1.68 vs. 10.14 ± 3.80 doses).

**Figure 2:**
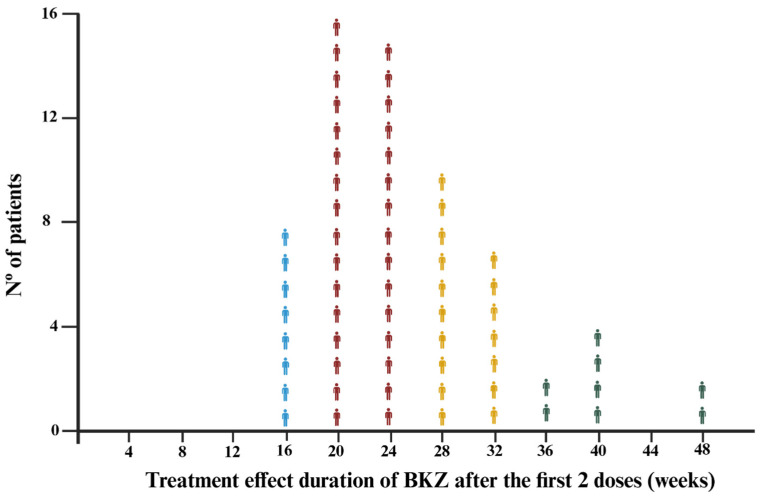
Dispersion plot showing the distribution of patients by treatment effect duration. In blue, patients with Short treatment effect duration (0–16 weeks) are represented; in red, those with Intermediate treatment effect duration (17–24 weeks); in yellow, Long treatment effect duration (25–32 weeks); in green, Very Long treatment effect duration (>33 weeks). **Legend:** BKZ: Bimekizumab. Nº: Number.

As shown in Table 2, patients were stratified according to treatment effect duration into four groups as shown in the dispersion plot (Figure 2): Short group: (0–16 weeks; *n* = 8), Intermediate (17–24 weeks; *n* = 31), Long (25–32 weeks; *n* = 17), and Very Long group (>33 weeks; *n* = 8). Although not statistically significant, patients in the Very Long group had the shortest psoriasis duration compared with the other groups (Very Long group: 11.9 ± 12.0 years vs. Long: 16.8 ± 10.7 years vs. Intermediate: 12.5 ± 12.6 years vs. Short: 21.3 ± 9.0; *p* = 0.056) and a higher prevalence of dyslipidemia (Very Long group: 75% vs. Long: 23.5% vs. Intermediate: 32.3% vs. Short: 50%; *p* < 0.076).

**Figure 3:**
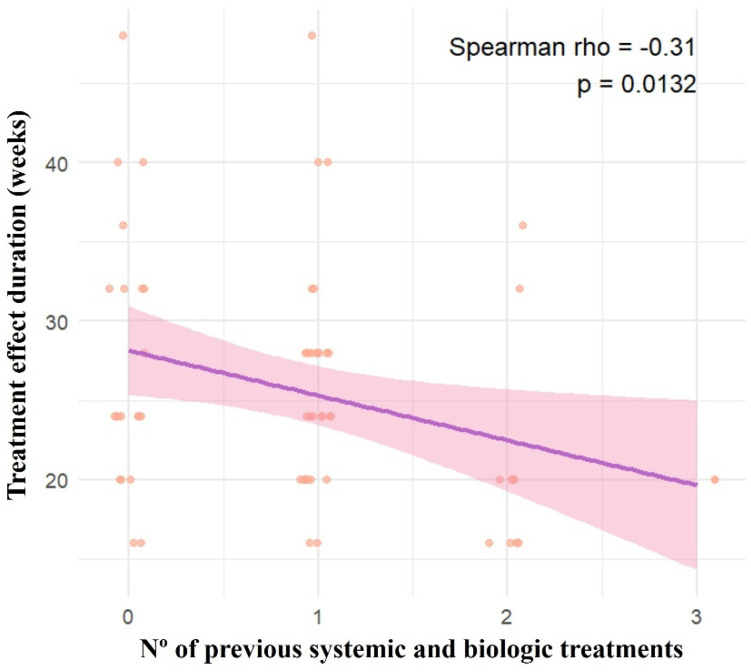
Correlation between treatment effect duration (weeks) and number of previous systemic and biologic treatments. Each patient is represented by a dot. **Legend**: Nº: Number. The purple line represents the fitted regression line (trend), while the shaded red area indicates the 95% confidence interval around the regression. Individual data points are shown as red circles.

In univariate analyses, treatment effect duration was only associated with the number of previous systemic treatments (*p* < 0.05), whereas age, BMI, baseline PASI, and psoriasis duration were not significantly associated with treatment effect duration (Figure 3).

In multivariable analysis, the number of previous systemic treatments remained independently associated with a shorter duration of clinical response (β = −0.76 months per prior treatment; *p* = 0.019). Subgroup analysis confirmed a progressive reduction in clinical response duration as the number of prior treatments increased. Conversely, the longest treatment duration responses were observed in systemic treatment-naïve patients.

## 4. Discussion

In this retrospective study of 64 patients with moderate-to-severe psoriasis, we confirm and extend our previous observations regarding the efficacy and safety of an off-label, as-needed dosing strategy for BKZ. Our findings demonstrate that a large treatment effect duration can be maintained with significantly reduced drug exposure over an extended follow-up period. Notably, over 90% of patients sustained PASI 90 responses using this individualized approach, receiving on average only one-third of the doses they would have received if the standard dosing label had been followed. The mean treatment effect duration interval following the first two BKZ doses (Week 0 and Week 4) was approximately 24 weeks. This duration is nearly three times longer than the maintenance dosing intervals reported in BKZ clinical trials. This observation aligns with the 24-week median time to the loss of PASI 90 response reported in the BE READY randomized withdrawal study [10]. In this trial, more than 90% of patients achieved PASI 90 at week 16, with responses maintained up to week 56 under fixed maintenance dosing regimens. Additionally, the median time to loss of response after treatment withdrawal was approximately 28–32 weeks, highlighting the durability of response even in the absence of continuous treatment. However, that trial explored sustained efficacy following complete treatment cessation after the 16-week induction period, whereas our real-world data suggest that a proactive as-needed dosing strategy following a 4-week (two injections) induction period could maintain optimal skin control (PASI 90) for similar durations of time. Our results suggest that dual IL-17A/F inhibition with BKZ may provide both rapid and sustained disease control, which may contribute to the prolonged treatment effect observed despite reduced dosing intensity. The ability to maintain high levels of efficacy with reduced and flexible dosing supports the use of response-guided strategies and underscores their potential contribution to personalized medicine.

A novel contribution of this study is the identification of clinical predictors for treatment effect duration. We found that the number of previous systemic and/or biologic treatments was the primary independent determinant of treatment effect duration. Specifically, patients with a lower prior treatment burden, and particularly those who were systemic- and biologic-naïve, exhibited significantly longer treatment durability. This is consistent with previous literature suggesting that prior exposure to biologics is associated with shorter times to relapse and reduced overall efficacy [11]. By contrast, increased BMI, often identified as a predictor of shorter treatment durability [9], was not correlated with treatment effect duration in our study. These findings suggest that dual IL-17A/F inhibition by BKZ may provide sufficient potency to overcome inflammatory challenges typically associated with obesity. Interestingly, patients with the longest treatment effect duration also exhibited the shortest psoriasis duration and a higher prevalence of dyslipidemia. While the latter finding should be interpreted cautiously due to the small sample size, the link between shorter psoriasis duration and sustained response supports the “window of opportunity” hypothesis in psoriasis [12].

Limitations of this work include its retrospective design, with the inherent risk of selection and information bias, as well as its case series-like nature, which limits statistical power and generalizability. In addition, the study was conducted in a specialized Psoriasis Unit, which may introduce a selection bias toward more treatment-adherent patients. The absence of a control group and the reliance on real-world data with potential heterogeneity in patient management, follow-up, and outcome assessment may also have influenced the results. Furthermore, assessment of disease recurrence between visits was partially based on patient-reported information, and PASI evaluations were not performed at strictly standardized time points due to the on-demand nature of follow-up visits. In addition, immunogenicity is a potential factor that could influence treatment effect duration. Although anti-drug antibodies (ADAs) were not measured in this study, phase III clinical trials have shown that approximately 45% of patients treated with BKZ develop ADAs [10,13,14]. As-needed dosing may allow drug levels to fall below therapeutic thresholds, potentially increasing the risk of immune recognition and secondary loss of response. Moreover, the use of telephone/email follow-up may have influenced the estimation of treatment effect duration. Future studies incorporating therapeutic drug monitoring will be important to clarify the role of immunogenicity in this setting.

In conclusion, this study demonstrates safe and effective as-needed dosing for BKZ in patients with psoriasis over time, with prior exposure to systemic or biologic therapy identified as the most important determinant of treatment effect duration. Therapeutic dose reduction strategies such as the one described here can optimize risk–benefit considerations in individual patients as well as reduce healthcare cares associated with long-term management of psoriasis.

## Figures and Tables

**Table 1:** Baseline demographics and disease characteristics.

	N = 64
Age (years)	42.4 ± 16.2
Sex, men (%)	42 (65.6%)
Age at the start of psoriasis (years)	27.7 ± 16.3
Psoriasis duration (years)	14.7 ± 11.8
BMI (kg/cm^2^)	25.1 ± 3.9
Psoriatic arthritis, *n* (%)	7 (10.9%)
Active smokers, *n* (%)	9 (14.1%)
Hypertension, *n* (%)	7 (10.9%)
Dyslipidemia, *n* (%)	24 (37.5%)
PASI	7.1 ± 4.8
BSA	7.2 ± 4.8
Nº of BKZ injections received	3.5 ± 1.7
Nº of BKZ injections expected (on-label)	10.9 ± 6.7
Treatment effect duration (months)	6.4 ± 1.9
Treatment effect duration (weeks)	25.6 ± 7.6
Previous systemic or biologic treatment, *n* (%)	38 (59.4%)
Previous systemic treatment, *n* (%)	21 (32.8%)
Previous biologic treatment, *n* (%)	20 (31.3%)

Data are presented as mean (SD) or median (IQR) for continuous measures, and *n* (%) for categorical measures. BKZ: Bimekizumab. BMI: Body Mass Index. BSA: Body Surface Area. Nº: Number. PASI: Psoriasis Area Severity Index.

**Table 2:** Patient characteristics according to treatment effect duration.

	Short N = 8	Intermediate N = 31	Long N = 17	Very Long N = 8	*p*-Value
Age (years)	50.1 ± 10.8	41.0 ± 18.3	39.1 ± 11.1	47.1 ± 20.2	0.3
Sex (men, %)	6 (75.0%)	20 (64.5%)	12 (70.6%)	4 (50.0%)	0.7
Psoriasis onset (years)	28.9 ± 16.0	28.4 ± 16.7	22.1 ± 11.8	35.4 ± 21.9	0.6
Psoriasis duration (years)	21.3 ± 9.0	12.5 ± 12.6	16.8 ± 10.7	11.9 ± 12.0	0.056
BMI (kg/cm^2^)	25.2 ± 2.3	24.6 ± 4.6	26.1 ± 3.5	24.6 ± 3.0	0.5
Psoriatic arthritis, *n* (%)	2 (25.0%)	2 (6.5%)	2 (11.8%)	1 (12.5%)	0.4
Active smokers, *n* (%)	0 (0.0%)	2 (6.5%)	5 (29.4%)	2 (25.0%)	0.076
Hypertension, *n* (%)	1 (12.5%)	4 (12.9%)	0 (0.0%)	2 (25.0%)	0.2
Dislipemia, *n* (%)	4 (50.0%)	10 (32.3%)	4 (23.5%)	6 (75.0%)	0.076
PASI	7.8 ± 6.2	7.0 ± 4.9	7.1 ± 4.4	6.8 ± 4.9	>0.9
BSA	6.9 ± 4.9	6.8 ± 4.9	7.8 ± 4.5	7.6 ± 5.9	0.8
Nº BKZ received	4.5 ± 2.8	3.6 ± 1.7	3.0 ± 1.0	3.0 ± 0.8	0.6
Nº BKZ if standard label was followed	15.3 ± 16.6	9.7 ± 3.9	10.6 ± 3.6	11.8 ± 3.3	0.4
Previous systemic or biologic treatment, *n* (%)	6 (75.0%)	20 (64.5%)	10 (58.8%)	2 (25.0%)	0.2
Previous systemic treatment, *n* (%)	3 (37.5%)	11 (35.5%)	6 (35.3%)	1 (12.5%)	0.7
Previous biologic treatment, *n* (%)	4 (50.0%)	11 (35.5%)	4 (23.5%)	1 (12.5%)	0.4

Data are presented as mean (SD) or median (IQR) for continuous measures, and *n* (%) for categorical measures. The four groups are defined as follows: Short (treatment effect duration from 0–16 weeks); Intermediate (treatment effect duration from 17–24 weeks); Long (treatment effect duration from 25–32 weeks); and Very Long (treatment effect duration from 33 onwards). BKZ: Bimekizumab. BMI: Body Mass Index. BSA: Body Surface Area. Nº: Number. PASI: Psoriasis Area Severity Index.

## Data Availability

The data underlying this article is available upon request from the corresponding author.
